# Rapid Detection of Estrogens in Cosmetics by Chemical Derivatization and Paper-Spray Ionization Mass-Spectrometry

**DOI:** 10.3390/molecules28031130

**Published:** 2023-01-23

**Authors:** Dongning Song, Song Yuan, Caiyu Zhang, Lin Luan, Yang Liu, Qingsheng Zhang

**Affiliations:** 1 National Institutes for Food and Drug Control, Beijing 102629, China; 2 NMPA Key Laboratory for Quality Research and Evaluation of Chemical Drugs, Beijing 102629, China

**Keywords:** paper-spray ionization mass spectrometry (PSI-MS), estrogens, cosmetics, chemical derivatization

## Abstract

Estrogens in personal care products are harmful to customers. Conventional methods such as HPLC and LC-MS require tedious sample pretreatment and long analytical time. Paper-spray ionization mass spectrometry (PSI-MS) is a powerful tool for the determination of compounds with little time and minimal pretreatment procedures. Since most estrogens show poor responses in PSI-MS, we developed a chemical derivatization and PSI-MS method to determinate three estrogens: estradiol, estriol and ethinyloestradiol with estradiol valerate as the internal standard (I.S.). After derivatization with 2-fluoro-1-methyl-pyridinium-p-toluene-sulfonate, the three estrogens could be quantified in seconds. This method showed good linearity in the range of 0.1~30 μg·mL^−1^, with R^2^ > 0.999. Their recovery results were all between 85%~115%. The limits of detection (LOD) were 0.04 μg·mL^−1^, 0.02 μg·mL^−1^ and 0.02 μg·mL^−1^ for estradiol, estriol and ethinyloestradiol respectively, which improved around 200, 2000, and 900 times compared to non-derivative PSI-MS. The method could quantitatively determine estrogens in cosmetics.

## 1. Introduction

Estrogen is a type of sex hormone which plays multiple functions in the body. Studies indicated that estrogens may have beneficial effects on skin functions such as preventing skin aging, increasing collagen elasticity and improving skin moisture [1,2,3], while estrogen also has strong physiological activities such as plumping body, accumulation of subcutaneous fat and promoting secondary sexual characteristics [4]. Thus, illegal addition of estrogens into cosmetics may cause severe side effects such as metabolism disorders of the human body, occurrence of breast cancer and endometrial hyperplasia [4,5]. China’s “Safety and Technical Standards for Cosmetics” [6] and the European Union Cosmetics Regulations [Regulation (EC) No 1223/2009] [7] both clearly stipulate that estrogens are banned from cosmetics. Hence, it is of great importance to develop a fast screening and quantitation method for the detection of illegally added estrogens in cosmetics. 

Several methods have been developed to detect estrogens in cosmetics including HPLC-UV [8,9], excitation–emission matrix fluorescence [4], GC-MS [10], HPLC-MS(/MS) [11,12,13], and immunoassays [14]. However, most of those methods need tedious pretreatments, such as solid phase extraction, liquid–liquid extraction, filtration etc. It is essential to develop a simple and effective method to deplete time-consuming pre-treatments. 

Mass spectrometry has been applied in many fields, such as proteomics [15], forensic medicine [16], biomedicine [17] and chemistry. But conventional mass spectrometry requires complex preprocessing to avoid contamination. Ambient ionization mass spectrometry (AMS) was first introduced in 2004 by Cooks et al. [18]. AMS can be operated in atmosphere environment and requires minimal pretreatment. Paper spray ionization mass spectrometry (PSI-MS) is one kind of AMS [19]. PSI-MS combines the advantages of AMS and electrospray ionization (ESI). It is suitable for rapid qualitative and quantitative analysis of compounds in various matrices [20]. PSI-MS has been used to detect many kinds of compounds, such as imatinib [19], amino acid neurotransmitters [21], extracellular vesicle [22], peroxide compounds [23]. 

**Figure 1:**
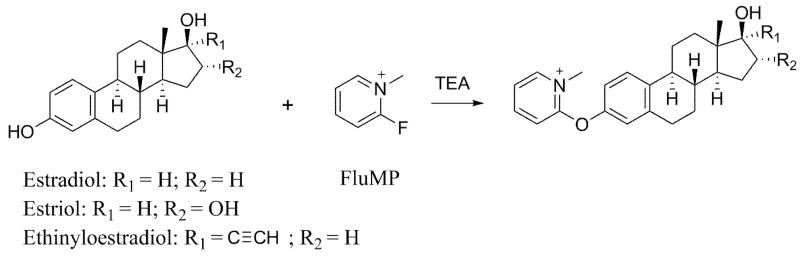
Derivative reaction of FluMP and estrogens.

Since most of estrogens contain only carbon, hydrogen and oxygen atoms, they show poor or no response in PSI-MS. Chemical derivatization is often utilized to modify the functional groups in target molecules and add charges to the final product. The added charges will effectively improve the ionization efficacy. Here, we developed a chemical derivatization and PSI-MS method for the rapid detection of estradiol, estriol and ethinyloestradiol in cosmetics based on Mukaiyama reaction (Figure 1). 2-fluoro-1-methyl-pyridinium-p-toluene-sulfonate (FluMP) was used as the derivative reagent. FluMP reacts with the three estrogens under the catalysis of triethylamine. This reaction finished within 60 s. After derivatization, the products could be easily analyzed by PSI-MS.

## 2. Results and Discussion

### 2.1. The Choose of Derivatization Reagents

Since estrogens show poor ionization efficacy under positive ESI mode, they generally give no response in PSI-MS. In order to improve detection sensitivity, chemical derivatization is adopted. There are several aspects to consider in choosing a suitable derivatization reagent. First, it should react with estrogens quickly and neatly. Second, the product should bear one or more positive charges which will help to improve sensitivity. Third, since the purpose of this study is to investigate the estrogens in cosmetics, the derivative reaction should not be interfered with other materials in cosmetics.

Betaine aldehyde chloride (BA) is a widely used derivative reagent to react with hydroxyl groups in molecules. When BA was mixed with estrogens, results showed that the reaction efficiency was low. This might be due to the poor reactivity of BA toward phenolic hydroxyl groups in estrogens [24]. Other derivative reagents such as dansyl chloride, pyridine-3-sulfonyl chloride and FluMP were previously reported to react with estrogens. [25,26]. Among them, FluMP can react with primary and secondary alcohols and phenols in complex mixtures [27,28]. After derivatization, a quaternary ammonium salt was introduced to the target estrogens, thus greatly improveing the response in mass spectrometry. FluMP also has been used to improve the detection limit of some phenolic compounds such as cannabinoids [29] and buprenorphine in MS [30]. So, in this study, FluMP was explored as the derivatization reagent for sensitive detection of estrogens.

### 2.2. The Optimization of Reaction Conditions

Since there are other compounds containing hydroxyl groups within cosmetics, it is necessary to use excess amount of FluMP to make sure all the estrogens are derivatized. It is found that 2000 times excess amount of FluMP can make sure all the estrogens are derivatized. After derivatization, 2 μL of final solution was deposited onto the triangular paper. The solution contained about 10 μg of FluMP. If the amount of FluMP was more than 10 μg, the tip of the paper might be blocked and electric discharge was observed occasionally at the tip of the paper.

**Figure 2:**
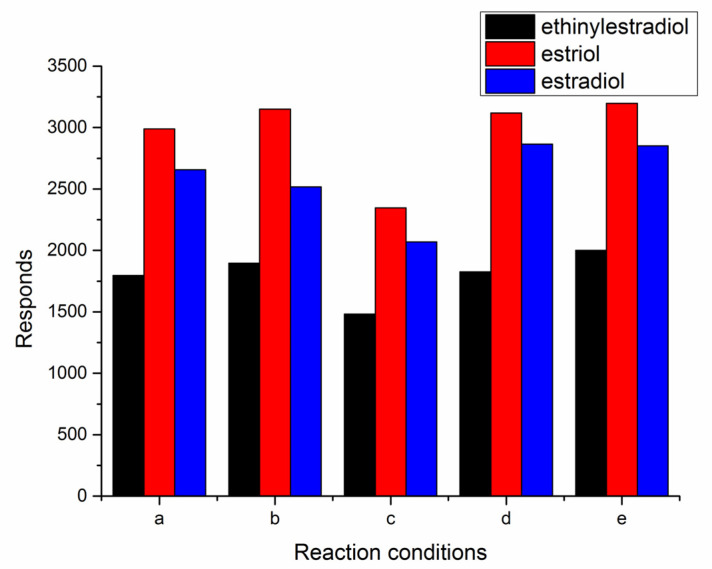
Influence of reaction conditions (a: without heat or ultrasound; b: heat for 10 min; c: heat for 20 min; d: ultrasound for 10 min; e: ultrasound for 20 min).

Temperature may also affect the efficacy of derivative reactions. After adding triethylamine, the final solution was heated at 60 °C for about 10 and 20 min. Results showed that high temperature had no obvious influence on the reaction. The final solution was also treated by ultrasound for 10 and 20 min. Data showed that ultrasound time had no effect on the final MS response (Figure 2). 

### 2.3. Optimization of Paper-Spray Mass Spectrometry Parameters

**Figure 3:**
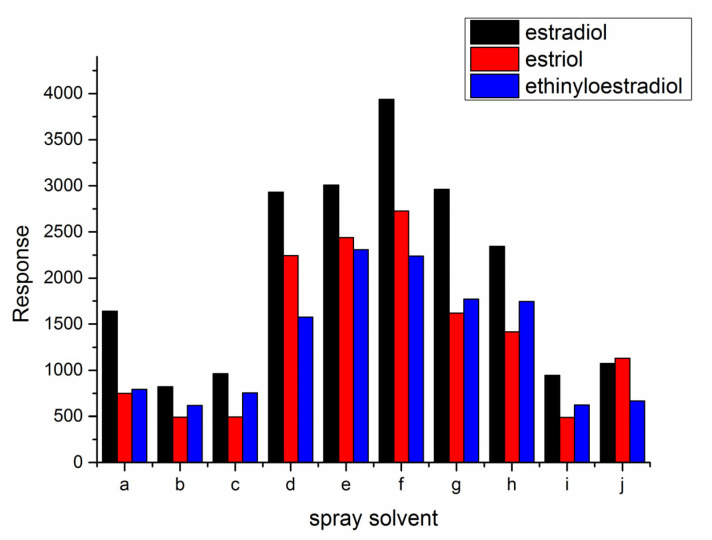
Influence of spray solvents: (a) ACN; (b) MeOH; (c) MeOH/H_2_O = 8/2; (d) ACN/H_2_O = 7/3; (e) ACN/H_2_O = 8/2; (f) ACN/H_2_O = 9/1; (g) ACN/H_2_O = 9.5/0.5; (h) 0.1% formic acid in ACN/H_2_O = 8/2; (i) 0.1% formic acid in ACN; (j) 0.1% formic acid in ACN/H_2_O = 5/5).

Spray solvent directly affects the response in PSI-MS. In this study, various solvent systems were explored, including water, acetonitrile and methanol etc. Best signal response was achieved when ACN/H_2_O = 9/1 (V/V) were used as the spray solvent (Figure 3). Methanol or formic acid in the solvent didn’t yield a high response. This may be related to the solubility of the derivatization products. The products are quaternary ammonium salts, which may have better solubility in acetonitrile and water than in methanol. Since the products are positively charged, the response value may be reduced by the ion suppression effect when the spray solvent contains formic acid.

The spray voltage and distance from the paper tip to the cone of mass spectrometry (d) were also optimized. The response reached the highest value when the spray voltage was 3.5 kV. Meanwhile, d less than 3 mm may cause electric discharge and no spray occurred when the d was more than 10 mm. Finally, the d was set at 5 mm.

### 2.4. Other Important Parameters

The time of the product was loaded on the paper will affect its adsorption, while the process of generating electric spray includes the process of desorption. Results showed that after the sample was loaded onto the paper, the response value will gradually reduce with the extension of time. Spray solvent should be added directly after loading the sample. 

### 2.5. Linearity, Lower Limits of Detection

**Figure 4:**
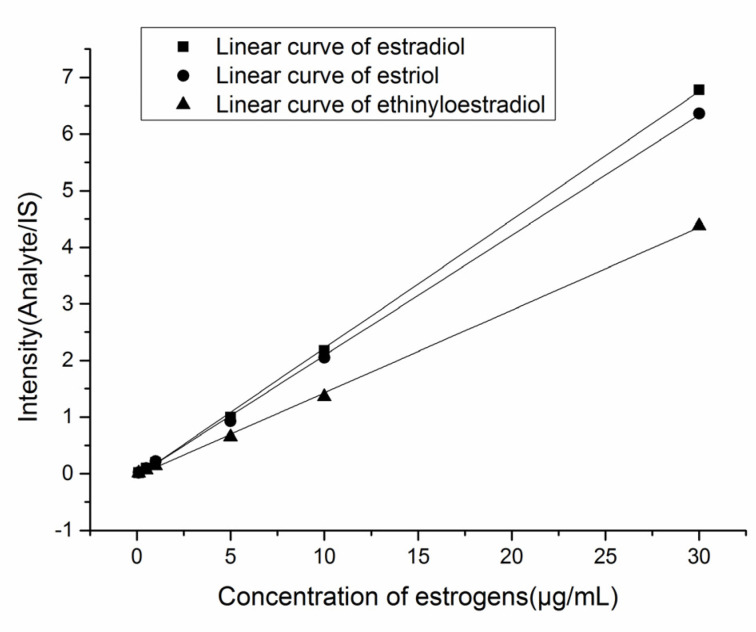
The linear curves of estradiol (Y = 0.2267x − 0.0482, R^2^ = 0.9995), estriol (Y = 0.2125x − 0.037, R^2^ = 0.9994), and ethinyloestradiol (Y = 0.1463x − 0.0344, R^2^ = 0.9991) after FluMP derivatization.

Calibration curves were constructed by plotting intensity of analyte to IS (Y) versus concentrations of analyte standards(X) at the highest peak point of TIC diagram (Figure 4). The lower limits of detection (LOD) were calculated by D = 3δ/S, where D represents LOD, δ represents the standard deviation of six injections of the blank solution and S represents the slope of the linearity. Their LOD were 0.04, 0.02, 0.02 μg·mL*^−^*^1^ respectively.

### 2.6. Recovery

To evaluate the accuracy of this method, recovery experiments were carried out. First, 2 mg blank sample was mixed with 20 μL linear standard solution with different concentrations (1 μg·mL*^−^*^1^, 10 μg·mL*^−^*^1^, 30 μg·mL*^−^*^1^). The solution was treated with ultrasound for 2 min. After 2 μL internal standard solution and 40 μL of FluMP solution (10 mg·mL*^−^*^1^ in acetonitrile) were added, the resulting solution was vortexed for 10 s, then 10 μL triethylamine was added. The final solution was vortexed for another 10 s then subjected to PSI-MS analysis immediately. Three replicates of per concentration sample, and their RSD were all less than 10%. The average recovery were 104.2% (1 μg·mL*^−^*^1^), 91.5% (10 μg·mL*^−^*^1^), 104.2% (30 μg·mL*^−^*^1^) for estradiol; 113.3% (1 μg·mL*^−^*^1^), 87.2% (10 μg·mL*^−^*^1^), 102.4 (30 μg·mL*^−^*^1^) for estriol and 112.9% (1 μg·mL*^−^*^1^), 101.7% (10 μg·mL*^−^*^1^), 105.1% (30 μg·mL*^−^*^1^) for ethinyloestradiol.

### 2.7. Detection Sample with Complex Matrix

To verify the practicability of this method, the quantified target compound was spiked into the complex matrix (cream containing octadecyl alcohol, liquid paraffin and glycerol), and then their content was determined by chemical derivatization and PSI-MS. Results showed that this method can precisely and quickly quantify the amount of added estrogens in cosmetics (Table 1).

Compared with directly monitoring estrogens by PSI-MS without chemical derivatization, method shown in this paper is more sensitive, with the sensitivity of estradiol, estriol, and ethinyloestradiol increased by 200, 2000, and 900 times respectively.

### 2.8. Estimation of Measurement Uncertainty (MU)

The estimation of MU was performed following EURACHEM/CITAC guide quantifying uncertainty in analytical measurement [31] with the data from in-house method validation. The MU was calculated by combining the overall accuracy and precision of nine experiments at three concentration levels (Equation (1)).

(1) $$U_{com}=\sqrt{U_{acc}{}^{2}+U_{pre}{}^{2}}$$

where *U_com_* is the combined uncertainty, *U_acc_* is the accuracy uncertainty (±20%) and *U_pre_* is the precision uncertainty (RSD of three replicates). 

And considering the coverage factor k = 2, the expanded MU was 0.1443, 0.1377 and 0.1475 for estradiol, estriol and ethinyloestradiol respectively. 

### 2.9. Estimation of Greenness Character

AGREE (Analytical GREEnness calculator) was used to estimate the greenness character of our experiment [32,33]. The parameters considered including the amounts and toxicity of reagents, waste generated and energy requirements etc. The final score is 0.94, which indicates that the experiment is environmentally friendly.

## 3. Materials and Methods

### 3.1. Reagents and Materials

Estradiol, Estriol, and Ethinyloestradiol were from National Institutes for Food and Drug Control (Beijing, China). Acetonitrile was purchased from Sigma-Aldrich (St. Louis, MO, USA). 2-fluoro-1-methyl-pyridinium-p-toluene-sulfonate was purchased form TCI chemicals (Shanghai, China). Triethylamine was from HUSHI (Shanghai, China). All the reagents were used directly without further purification.

### 3.2. Instrument

All experiments were carried out with an Agilent 1290 HPLC coupled with 6470 triple quadruple mass spectrometer (Palo Alto, CA, USA). Data were acquired and processed by Agilent MassHunter Workstation 10.1 (Palo Alto, CA, USA). HB-Z303-1AC High-voltage DC Power supply (Tianjin, China) and KQ-500DA CNC ultrasonic cleaner (Kunshan, China) were used. Grade 1 chromatographic paper was from Whatman (Stevenage, UK).

### 3.3. Sample Preparation

Mixed standard stock solution (50 μg·mL^−1^): Approximately 2 mg estradiol, 2 mg estriol and 2 mg ethinyloestradiol were dissolved in 10 mL acetonitrile. Then 5 mL of the resulting solution was transferred to a 20 mL volumetric flask and was diluted to volume with acetonitrile.

Linear standard solutions: Mixed standard solution was diluted to 0.1, 0.5, 1, 5, 10 and 30 μg·mL^−1^ with acetonitrile.

Internal standard solution: After 5 mg estradiol valerate was transferred to a volumetric flask, 50 mL acetonitrile was used to dissolve estradiol valerate.

### 3.4. Derivative Reaction

Derivatization of linear standard solutions: After 2 μL internal standard solution and 20 μL linear standard solution were mixed, 40 μL of FluMP solution (10 mg·mL^−1^ in acetonitrile) was added to the solution. The resulting solution was vortexed for 10 s, then 10 μL triethylamine was added. The final solution was vortexed for another 10 s then subjected to PSI-MS analysis immediately.

Derivatization of samples: After 2 mg sample was added into 20 μL acetonitrile and was treated by ultrasound for 2 min, 2 μL internal standard solution and 40 μL of FluMP solution (10 mg·mL^−1^ in acetonitrile) were then added. The resulting solution was vortexed for 10 s, then 10 μL triethylamine was added. The final solution was vortexed for another 10 s then subjected to PSI-MS analysis immediately.

### 3.5. Mass Spectrometry Parameters

**Figure 5:**
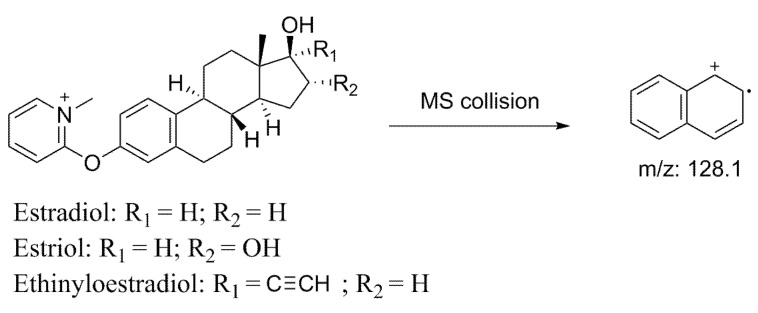
Fragmentation path of estradiol, estriol and ethinyloestradiol.

The filter paper was cut into isosceles triangle (5 × 13 cm) and the bottom side was clipped by a copper clamp which is connected to a voltage source. The voltage was 3.5 kV. The paper tip was positioned approximately 5 mm from the mass spectrometer inlet. A volume of 20 μL of acetonitrile:H_2_O = 9:1 was used as the spray solvent. The derivatives of three estrogens were all detected in positive multiple reaction monitoring (MRM) mode. Their parent ions and daughter ions were 364.2→128.1; 380.2→128.1; 388.2→128.1 for estradiol, estriol and ethinyloestradiol respectively (Figure 5). The collision energy was 65 eV. Both the gas and sheath temperature were 100 °C.

### 3.6. PSI Analysis

A volume of 2 μL derivative reaction solution was deposited onto the triangular paper and direct current power was applied. Then spray solvent was added to allow the spray desorption.

## 4. Conclusions

In this article, a chemical derivatization and PSI-MS method was established to detect estrogens that may exist in cosmetics. Meanwhile, this strategy has several aspects to improve, such as, (1) FluMP reacts well with phenol hydroxyl but has poor effect on alcohol hydroxyl, it is better to find a more universal derivative reagent; (2) for compounds that have functional groups other than -OH, different derivative reagents may required. In the future, more universal derivative reagents which are suitable for in-situ PSI experiments will be studied to further reduce waste solvents and shorten time. Application of this chemical derivatization and PSI-MS may also be studied to characterize estrogens in blood or urine. 

Nontheless, this method has high sensitivity and does not require much pre-treatment. After derivatization, compounds’ responses improve 200–2000 times compared to non-derivatization PSI-MS. This method renders a new strategy for fast screening of low response compounds on PSI-MS in complex matrix. It might be further deployed to environmental or biological samples.

## Figures and Tables

**Table 1:** The results of detection samples with complex matrix.

Estradiol			Estriol			Ethinyloestradiol		
Added (μg/g, N = 3)	Found (μg/g, N = 3)	RSD% (N = 3)	Added (μg/g, N = 3)	Found (μg/g, N = 3)	RSD% (N = 3)	Added (μg/g, N = 3)	Found (μg/g, N = 3)	RSD% (N = 3)
10	10.42	8.28	10	11.33	3.30	10	11.29	5.67
100	91.54	5.75	100	87.20	3.17	100	101.73	2.73
300	312.64	5.15	300	307.30	5.19	300	315.36	9.47

## Data Availability

The data presented in this study are available on request from the corresponding author.
